# Azosemide is more potent than bumetanide and various other loop diuretics to inhibit the sodium-potassium-chloride-cotransporter human variants hNKCC1A and hNKCC1B

**DOI:** 10.1038/s41598-018-27995-w

**Published:** 2018-06-29

**Authors:** Philip Hampel, Kerstin Römermann, Nanna MacAulay, Wolfgang Löscher

**Affiliations:** 1 Department of Pharmacology, Toxicology and Pharmacy, University of Veterinary Medicine Hannover, Hanover, Germany; 2 Center for Systems Neurosciences Hannover, Hanover, Germany; 3 Department of Neuroscience, University of Copenhagen, Copenhagen, Denmark

## Abstract

The Na^+^–K^+^–2Cl^−^ cotransporter NKCC1 plays a role in neuronal Cl^−^ homeostasis secretion and represents a target for brain pathologies with altered NKCC1 function. Two main variants of NKCC1 have been identified: a full-length NKCC1 transcript (NKCC1A) and a shorter splice variant (NKCC1B) that is particularly enriched in the brain. The loop diuretic bumetanide is often used to inhibit NKCC1 in brain disorders, but only poorly crosses the blood-brain barrier. We determined the sensitivity of the two human NKCC1 splice variants to bumetanide and various other chemically diverse loop diuretics, using the *Xenopus* oocyte heterologous expression system. Azosemide was the most potent NKCC1 inhibitor (IC_50_s 0.246 µM for hNKCC1A and 0.197 µM for NKCC1B), being about 4-times more potent than bumetanide. Structurally, a carboxylic group as in bumetanide was not a prerequisite for potent NKCC1 inhibition, whereas loop diuretics without a sulfonamide group were less potent. None of the drugs tested were selective for hNKCC1B vs. hNKCC1A, indicating that loop diuretics are not a useful starting point to design NKCC1B-specific compounds. Azosemide was found to exert an unexpectedly potent inhibitory effect and as a non-acidic compound, it is more likely to cross the blood-brain barrier than bumetanide.

## Introduction

The Na^+^–K^+^–2Cl^−^ cotransporter NKCC1 (encoded by *SLC*1*2A2*) plays an important role in Cl^-^ uptake in neurons both in developing brain and in adult sensory neurons^1,2^. Alterations in the expression and function of NKCC1 have been implicated in several brain disorders, including neonatal seizures, epilepsy, autism, cerebral edema following ischemic and traumatic brain injury, and in chronic and acute pain^1,3–5^. Indeed, experimental studies indicate beneficial modulation of NKCC1 in models of these pathologies. Currently, bumetanide, a 5-sulfamoylbenzoic acid derivative loop diuretic, is the only drug that is used to inhibit neuronal NKCC1 in neurological or psychiatric disorders^5^. The potent diuretic effect of bumetanide is mediated by inhibition of NKCC2 (*SLC12A1*) in the thick ascending limb of the loop of Henle (TALH)^6^. Bumetanide reaches its target (NKCC2) in the kidney by active renal uptake mediated by organic anion transporters (OATs [*SLC22A*])^7,8^. However, in most other tissues, including the brain, only very low bumetanide concentrations are reached^9–11^. The low brain concentrations of bumetanide obtained after systemic administration are thought to result from its high ionization (>99%) at physiological pH and its high plasma protein binding (>95%), which restrict brain entry by passive diffusion, as well as active efflux transport at the blood-brain barrier(BBB)^9,10^. The poor brain penetration of bumetanide is a likely explanation for its controversial efficacy in the treatment of brain diseases^11–13^.

We have recently reported that BBB permeant prodrugs of bumetanide may provide a strategy to overcome the poor brain penetration of this drug^14,15^. However, this strategy would not resolve potential problems associated with NKCC1 inhibition in other tissues, including its ototoxicity in the inner ear^13,16,17^. In cells and tissues from different mammalian species, including rodents and humans, two major, alternatively spliced RNA variants of NKCC1 have been identified: a full-length NKCC1 transcript (NKCC1A) and a shorter splice variant (NKCC1B); both of which form functional cotransporters when expressed in heterologous systems^18^, and vary in their distribution in humans and other mammals^2,18–20^. Both NKCC1A and NKCC1B transcripts are expressed in the human brain, but the ratio of hNKCC1B to hNKCC1A expression is significantly higher in the brain, when compared with other tissues^18^. The functional significance and pharmacological sensitivity of the two main NKCC1 splice to various inhibitors is unknown. Developing pharmacological tools selectively targeting each variant has a potential impact on elucidating the function of these variants, as well as possible therapeutic implications for the treatment of various neurological and psychiatric disorders. In addition to hNKCC1A and hNKCC1B, two other splices variants of hNKCC1 have been reported recently^20^, but their tissue distribution and function are not yet known.

In the present study, we determined the sensitivity to bumetanide and other loop diuretics of the two main hNKCC1 splice variants, using the *Xenopus* oocyte heterologous expression system and radiolabeled rubidium (^86^Rb^+^) to measure NKCC-mediated ion flux. Our study had two major aims. First, we wanted to characterize the pharmacological profile of the hNKCC1A and hNKCC1B by using loop diuretics from different structural groups (see Results), thus allowing structure-activity analyses. Second, because to the best of our knowledge, except for bumetanide and furosemide, none of the chemically diverse loop diuretics used here have previously been tested for inhibitory effects on hNKCC1, we wanted to evaluate whether any of these drugs inhibit hNKCC1 with similar or higher potency than bumetanide, but, based on structure, physicochemical properties, and pharmacokinetics may have advantages vs. bumetanide for treatment of brain diseases with abnormal cellular chloride homeostasis.

## Materials and Methods

### Drugs

All chemicals were purchased from Sigma Aldrich Chemie GmbH (Schnelldorf, Germany), unless otherwise stated. Torasemide came from Absource Diagnostics GmbH (Munich, Germany), azosemide from MolPort SIA (Riga, Latvia), and tripamide from Shanghai Laihao Trade Co., Ltd (Shanghai, China). All NKCC inhibitors were dissolved in dimethyl sulfoxide (DMSO; <1% DMSO in final solution). Lipophilicity (log*P*) and acidic dissociation constant (p*K*a) of some of the drugs were taken from the DrugBank database (https://www.drugbank.ca/drugs/).

### Oocyte preparation and NKCC1 protein expression

Oocytes from *Xenopus laevis* were obtained from our own frogs (Laboratory Animal Science, Centre for Medical Sciences, Hannover Medical School, Germany). The surgical removal and preparation of defolliculated oocytes was performed as previously described^21^. Experiments were performed according to the European Union Guidelines for Animal Welfare (Directive 210/63/EU) and the German Law on Animal Protection (“Tierschutzgesetz”). Ethical approval for the study was granted by an ethical committee (according to §15 of the Tierschutzgesetz) and the governmental agency (Lower Saxony State Office for Consumer Protection and Food Safety; LAVES) responsible for approval of animal experiments in Lower Saxony (reference number for this project 15/1825). All efforts were made to minimize both the suffering and the number of animals.

Human NKCC1A (NM_001046, obtained from Prof. Biff Forbush, Yale School of Medicine, CT) and NKCC1B (prepared by site-directed mutagenesis from hNKCC1A as described below) were subcloned into the oocytes expression vector pXOOM, linearized downstream from the poly-A segment, and *in vitro* transcribed using T7-mMessage Machine according to manufacturer’s instructions (Ambion, Austin, TX). cRNA was then extracted with MEGAclear (Ambion, Austin, TX) and micro-injected into defolliculated *Xenopus laevis* oocytes (25 ng RNA/oocyte). The oocytes were kept in Kulori medium (in mM: 90 NaCl, 1 KCl, 1 CaCl_2_, 1 MgCl_2_, 5 Hepes, pH 7.4, 182 mOsm) for 5–6 days at 19 °C prior to experiments.

The difference between hNKCC1A (NM_001046) and hNKCC1B (NM_001256461) is 48 base pairs^18^. To obtain the cDNA encoding NKCC1B, two rounds of PCR (each deleting 24 base pairs from the sequence) was carried out with the following primer sets: shNKCC1A-B, 1–24: aaaaaccaattacacac.actgcaactcaaccact; ahNKCC1A-B, 1–24: agtggttgagttgcagtgtgtgtaattggttttt and shNKCC1A-B, 25–48: aaaaaccaattacacac_aaagaatccaaaggccc; ahNKCC1A-B, 25–48: gggcctttggattcttt gtgtgtaattggttttt. The entire sequence of the final construct was verified against hNKCC1B (NM_001256461).

### NKCC1A and NKCC1B activity assay

To activate NKCC1A and NKCC1B prior to the ^86^Rb^+^ uptake measurements aimed at measuring NKCC-mediated fluxes, hNKCC1A- or hNKCC1B-expressing oocytes or uninjected control oocytes (4–13 oocytes per well) were pre-incubated for 30 min at room temperature in a K^+^-free solution (containing in mM: 5 choline chloride, 95 NaCl, 1 MgCl_2_, 1 CaCl_2_, 10 Hepes, pH 7.4, 207 mOsm), which causes shrinkage of the oocyte and thus activation of NKCC1A^22^. To measure K^+^ influx, oocytes were exposed to an isosmotic test solution in which KCl (5 mM) replaced choline chloride, and 2 μCi/mL ^86^Rb^+^ (NEZ072, PerkinElmer, Rodgau, Germany) were included as a tracer for K^+^. Osmolarities of the test media were verified by using an osmometer (Type 15, Löser; Berlin, Germany). Different concentrations of drugs or control vehicle (≤1%; ensuring equal exposure to relevant drug solvent of all tested oocytes in the given experiment) were added to the test solution. The uptake assay was performed at room temperature for 5 min, which has been demonstrated to be within the linear phase of K^+^ uptake^22^. The influx experiments were terminated by 3 rapid washes in ice-cold ^86^Rb^+^-free assay solution after which the oocytes were individually dissolved in 50 µl 10% sodium dodecyl sulfate in scintillation vials. The radioactivity was determined by liquid scintillation β-counting with Aquasafe 300 Plus scintillation cocktail (Zinsser Analytic GmbH, Frankfurt, Germany) using a Microbeta Trilux (Perkin Elmer). hNKCC1-mediated K^+^ uptake was assessed as ([flux_NKCC1-expressing oocytes_ in presence of x µM drug]−[flux_uninjected oocytes_ in presence of x µM drug]), in order to correct for endogenous NKCC activity. All experiments were repeated at least three times (range 3–7) with 4–20 (in most experiments at least 10) oocytes per drug concentration per experiment.

### Western blotting of hNKCC1A and hNKCC1B in injected oocytes

Five to six days after micro-injection of hNKCC1A or hNKCC1B cRNA, single oocytes and respective uninjected oocytes were homogenized on ice by trituration through 23G cannulas in 20 µl lysis buffer per oocyte (20 mM Tris-HCl, 140 mM NaCl, 2% (v/v) Triton X-100) supplemented with complete protease inhibitor (Roche, Mannheim, Germany). Samples were stored at −20 °C until Western Blot analysis.

Protein concentrations were determined by using the Pierce BCA Protein Assay kit (Thermo Fisher Scientific, Darmstadt, Germany). 20 µg of total protein per sample were separated on 10% polyacrylamide gels under reducing conditions and transferred to PVDF membranes which were blocked for 2 h at room temperature (RT) in 5% nonfat milk in phosphate buffered saline supplemented with Tween-20 (PBST: 137 mM NaCl, 2.7 mM KCl, 4.3 mM Na2HPO4, 1.4 mM KH2PO4, pH 7.3, 0.05% (w/v) Tween-20). Membranes were cut at 70 kDa, incubated overnight with rabbit polyclonal anti-NKCC1 antibody (ab59791, Abcam, Cambridge, UK) 1:1000 and rabbit polyclonal anti-β-actin 1:5000 (Sigma-Aldrich, Taufkirchen, Germany) respectively, in 2% milk in PBST shaking at 4 °C and washed three times for 10 min in PBST. Ab59791 binds to the N terminal region of hNKCC1 and reacts with NKCC1 from human, mouse and rat. As the immunogen sequence is to 95% homologous to *Xenopus laevis* Nkcc1, it is likely to cross-react with the endogenous protein in frog oocytes (see Results). Secondary antibody goat anti-rabbit-HRP 1:1000 (Dako, Hamburg, Germany) was incubated for 1 h in 2% milk in PBST at RT and washed three times for 10 min in PBST. Proteins were detected by enhanced chemiluminescence using SuperSignal West Femto Chemiluminescent Substrate (Thermo Fisher Scientific) and the Chemidoc™ XRS Imager (Bio-Rad Laboratories, Munich, Germany). Relative protein expressions were quantified densitometrically (after subtracting background staining) with Quantity One 1-D Analysis software (Bio-Rad Laboratories) and calculated by normalization to the reference signals of β-actin with Microsoft® Excel 2010 (Microsoft Corporation, Redmond, USA) and GraphPad Prism 7.0 software (GraphPad, San Diego, CA, USA).

### Data analysis and statistical methods

Sigmoidal curves were fitted to the data for determination of the IC_50_ value for loop diuretics using GraphPad Prism 7.0, according to a dose-response inhibition curve with log (inhibitor vs. response, variable slope; Y = Min + (Max-Min)/(1 + 10^((LogIC_50_-X)*HillSlope)) and for experiments with fewer concentrations assuming the curves would reach complete inhibition (going from 100% to 0 according to a dose-response inhibition curve with log(inhibitor) vs. normalized response, variable slope; Y = 100/(1 + 10^(X-LogIC_50_)). IC_50_ values were obtained from each individual experiment, and averaged (using the median) across all experiments (at least 3) with the given drug to obtain an average IC_50_. Median rather than mean values were used because oocyte data on ^86^Rb^+^ uptake measurements were not normally distributed.

Although different batches of oocytes express minor differences in hNKCC1A or B levels, we did not observe any trends towards batch-specific differences in the IC_50_s of loop diuretics; therefore differences in expression levels should not affect the obtained values (with the exception of ethacrynic acid; see Results). All experiments are shown with median and interquartile range (or 95% confidence limits), whereas IC_50_s are presented as medians and ranges, in which the range was calculated from the number of experiments (3–7) per compound. Significance of differences in transport activity of hNKCC1A or hNKCC1B expressing oocytes vs. uninjected oocytes were analyzed by the Kruskal-Wallis test followed by Dunn’s multiple comparisons test. The Mann-Whitney test was used to calculate whether drug effects on hNKCC1A and hNKCC1B differed significantly. A P < 0.05 was considered significant.

### Data availability

The datasets generated and analyzed during the current study are available from the corresponding author on reasonable request.

## Results

### Structural diversity of loop diuretics used for studying the pharmacological sensitivity of hNKCC1A vs. hNKCC1B

**Figure 1:**
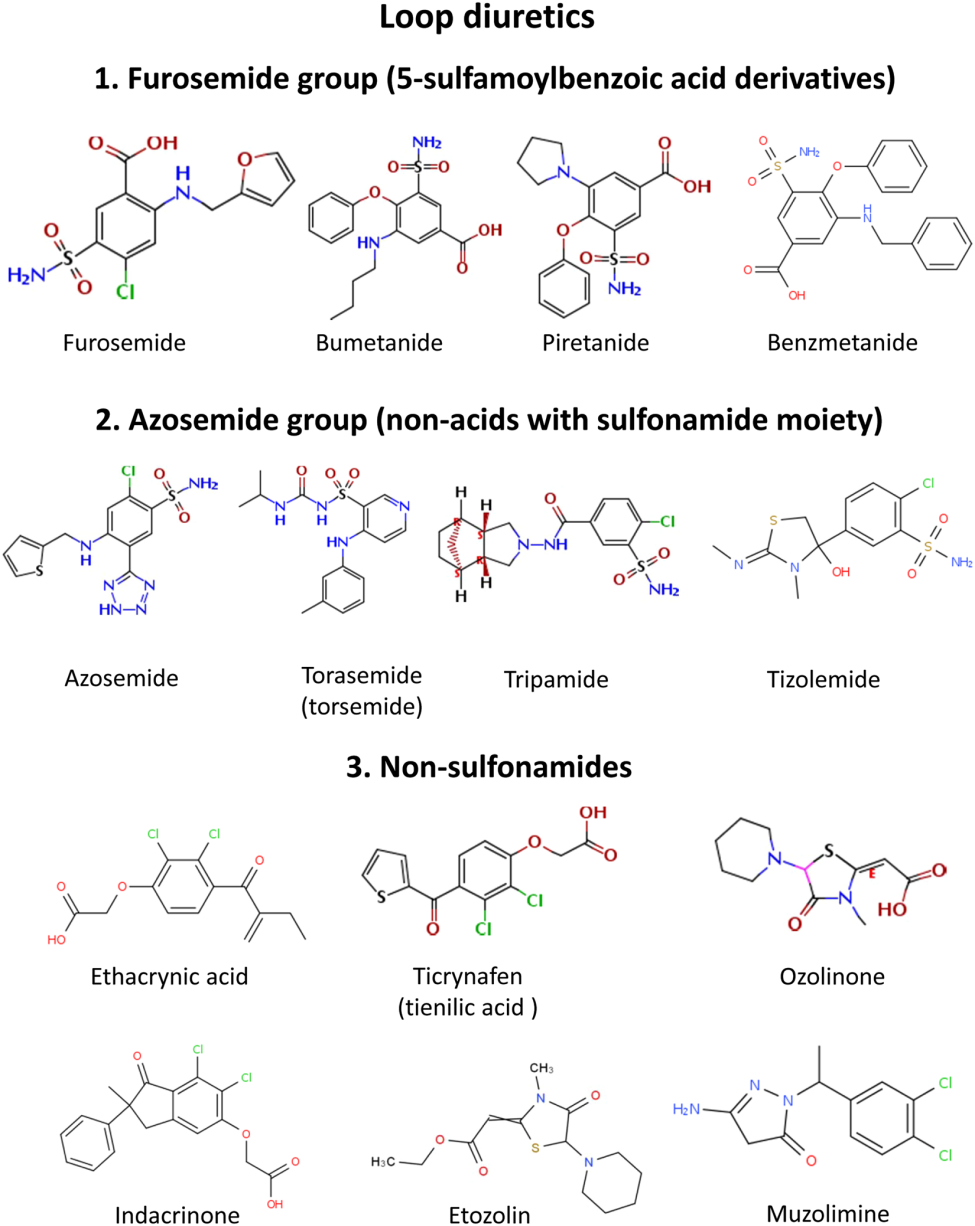
Chemical structures of clinically approved loop diuretics. According to their structures, the drugs were assigned to three groups. Several members of each group were tested for their potency to inhibit hNKCC1A vs. hNKCC1B.

As shown in Fig. 1, loop diuretics with different chemical structures can be classified into three groups; (1) 5-sulfamoylbenzoic acid derivatives furosemide, bumetanide, piretanide and benzmetanide; (2) the azosemide group, i.e., non-acids with a sulfonamide moiety, such as azosemide (axosemide), torasemide (torsemide), tripamide, and tizolemide, and (3) non-sulfonamides of different structure, which include both carboxylic acids like ethacrynic acid, ticrynafen (tienilic acid), indacrinone, and ozolinone, and non-acids such as etozolin and muzolimine. All these chemically different loop diuretics act by inhibiting NKCC2 in the TALH but differ in diuretic potency and efficacy, duration of the diuretic effect, pharmacokinetic properties, and safety profile^23–27^. The diverse chemical structures of the loop diuretics included in our experiments allowed structure-activity relationships between the inhibition of hNKCC1A vs. hNKCC1B. We were particularly interested in non-acidic compounds because they are likely to cross the BBB by passive diffusion, to a greater extent than bumetanide does, and could therefore be advantageous as NKCC1 inhibitors for potential therapeutic effects in brain diseases.

In the present study we used 8 loop diuretics of the 3 structurally different groups shown in Fig. 1 to evaluate structure-activity relationship of inhibition of hNKCC1A vs. hNKCC1B. For comparison with loop diuretics, two sulfonamide diuretics that act at other sites in the kidney, i.e., xipamide (4-chloro-5-sulfamoyl-2′,6′-salicyloxylidide) and glibenclamide (5-chloro-N-(2-{4-[(cyclohexylcarbamoyl)sulfamoyl] phenyl}ethyl)-2-methoxybenzamide), were included in our experiments as controls.

### Expression and transport activity of hNKCC1A and hNKCC1B vs. endogenous NKCC1 in injected and uninjected *Xenopus laevis* oocytes

**Figure 2:**
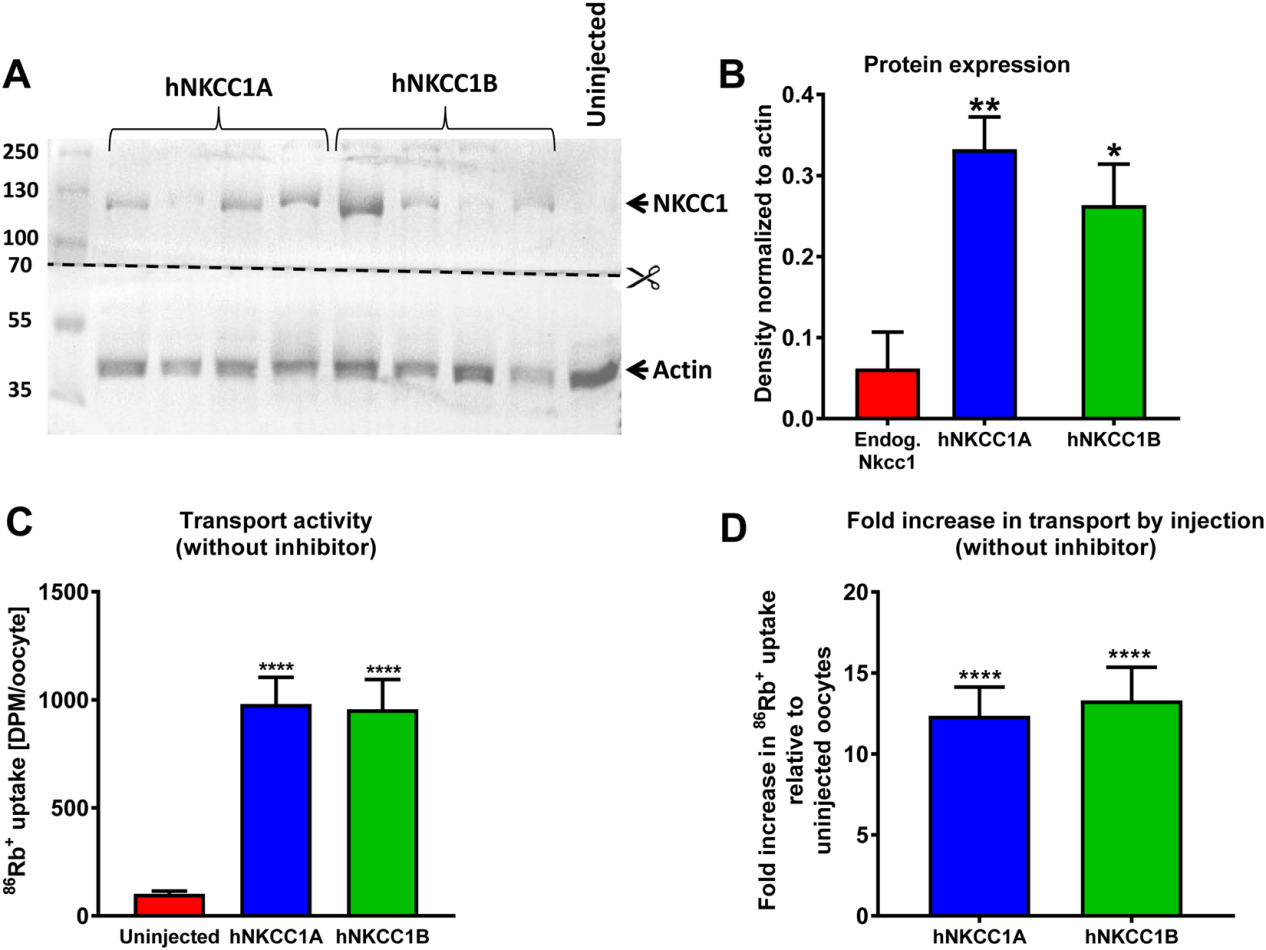
Heterologous expression of hNKCC1A and hNKCC1B in *Xenopus laevis* oocytes, determined by Western blotting, and the effect on transport activity, measured by ^86^RB^+^ uptake in the absence of NKCC1 inhibitors. (**A**) Representative Western blots of hNKCC1A and hNKCC1B protein expression in injected oocytes as well as endogenous NKCC1 in uninjected oocytes determined with the anti-NKCC1 antibody ab59791. The membrane was cut at 70 kDa in order to perform anti-NKCC1 and anti-β-actin immunostainings in separate containers (for more details see Suppl. Figure 1). In **B–D**, data are shown as means ± SEM. Significant differences to uninjected controls is indicated by asterisks (*P < 0.05; *P < 0.01; ****P < 0.0001). (**B**) Quantitative data on protein expression of endogenous NKCC1, hNKCC1A, and hNKCC1B after normalization to the reference signals of ß-actin. Data are from 4 (uninjected controls) and 7 (hNKCC1A, hNKCC1B) independent experiments, with 1–4 oocytes per experiment. Protein expression of hNKCC1A vs. hNKCC1B was not significantly different. (**C**) Absolute values showing ^86^RB^+^ uptake in uninjected control oocytes (reflecting endogenous NKCC activity) vs. uptake in oocytes expressing either hNKCC1A or hNKCC1B. (**D**) Fold increase in ^86^RB^+^ uptake by oocytes expressing either hNKCC1A or hNKCC1B compared to uninjected oocytes. Data in (**C**) and (**D**) are from 22 (uninjected controls), 21 (hNKCC1A) and 20 (hNKCC1B) independent experiments, with 4–20 oocytes per experiment.

The inhibitory potency of several diuretics on the transport activity of hNKCC1A or hNKCC1B was determined upon heterologous expression of hNKCC1 in *Xenopus laevis* oocytes. *Xenopus* oocytes have low endogenous expression of NKCC1^28^, which therefore does not contribute greatly to the results obtained with the heterologously expressed NKCC1 isoforms. This was verified by Western blot in the *Xenopus laevis* oocytes used here, showing a low expression (Fig. 2A,B) and transport activity (Fig. 2C) of endogenous NKCC1.

Following injection of hNKCC1A and hNKCC1B, NKCC1 protein expression in the oocytes significantly increased as expected (Fig. 2B). No size difference was seen for NKCC1B versus NKCC1A in the Western blots (Fig. 2A), because with 16 amino acids missing in NKCC1B the size difference is very small. However, as described in the Methods section, the identity of NKCC1B was proven by sequencing the cDNA construct. Heterologous expression of hNKCC1A in *Xenopus* oocytes increased the ^86^Rb^+^ uptake in median 10.3 fold (interquartile range: 5.68–15.9; 21 independent experiments) compared to the uninjected oocytes (Fig. 2D). Respective figures for hNKCC1B were 9.3 fold (interquartile range: 6.44–18.7; 20 independent experiments) (Fig. 2D), indicating that both splice variants of hNKCC1 transported ^86^Rb^+^ with similar efficacy.

### Inhibitory potency of bumetanide in the hNKCC1A/B oocyte assay

**Figure 3:**
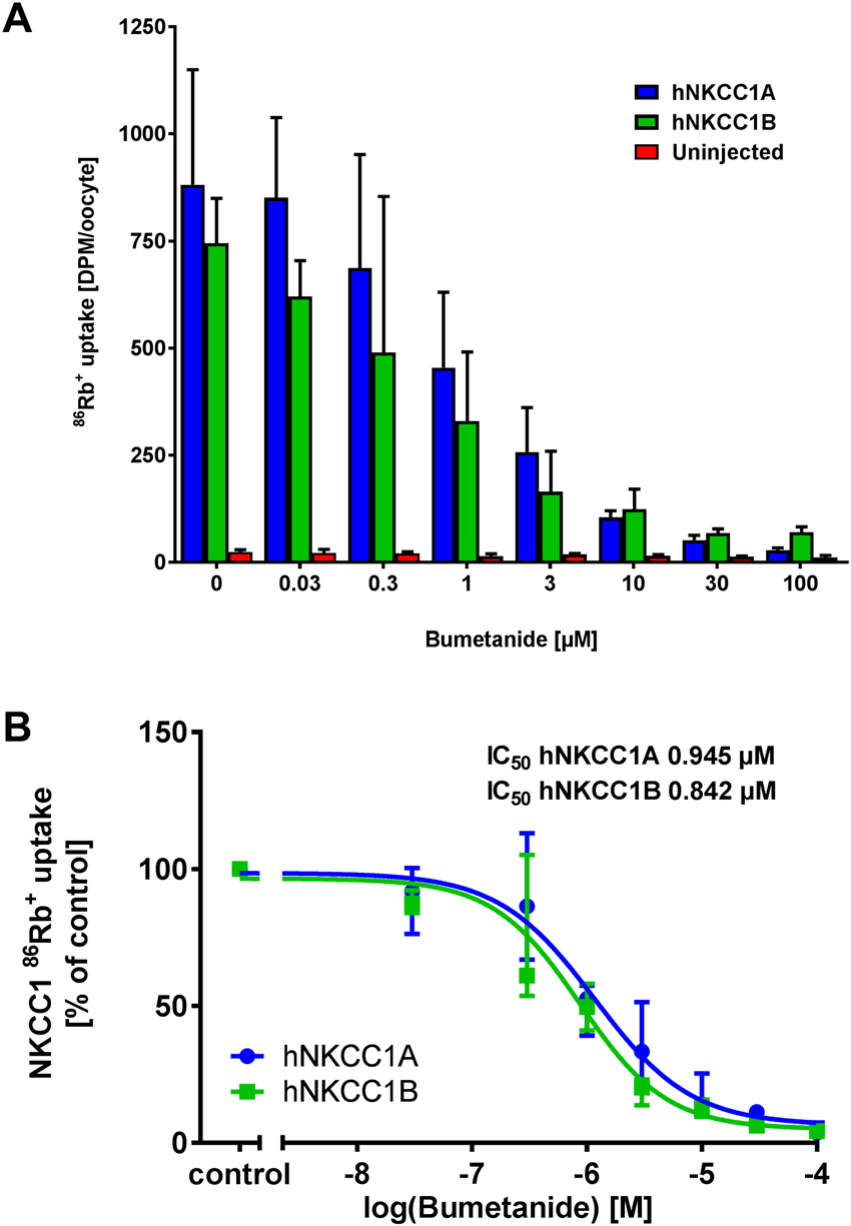
Effect of bumetanide on hNKCC1A- and hNKCC1B-mediated ^86^Rb^+^ uptake in *Xenopus* oocytes. (**A**) A representative experiment demonstrating the inhibitory effect of 0.03–100 µM bumetanide on ^86^Rb^+^ uptake (in CPM, counted for 10 minutes) in hNKCC1A- and hNKCC1B-expressing oocytes and on batch-matched uninjected oocytes. Data are shown as median of n = 5–10 oocytes per condition and error bars illustrate the interquartile range. (**B**) Dose-inhibition curve of bumetanide on hNKCC1A- and hNKCC1B-mediated ^86^Rb^+^ uptake (corrected for endogenous NKCC contribution in uninjected oocytes) normalized to control (0 µM bumetanide) and averaged across 7 experiments, with the IC_50_ calculated from each individual experiment prior to averaging.

Bumetanide inhibited the ^86^Rb^+^ uptake in oocytes expressing NKCC1A or NKCC1B in a dose-dependent manner (Fig. 3A, gray bars). The ^86^Rb^+^ uptake was reduced to the level observed in uninjected oocytes at bumetanide concentrations between 30 and 100 µM, as previously observed for hNKCC1A^29^. The endogenous contribution to NKCC-mediated ^86^Rb^+^ uptake in the uninjected oocytes was very small, as illustrated by bumetanide inhibition of the ^86^Rb^+^ uptake in uninjected oocytes (Fig. 3A, black bars), in agreement with earlier observations^28^. To deduct the contribution from the endogenous NKCC1, bumetanide-sensitive ^86^Rb^+^ uptake in uninjected oocytes was established in parallel and subtracted from the ^86^Rb^+^ uptake obtained in the hNKCC1A/B-expressing oocytes prior to determination of the IC_50_ for hNKCC1A or hNKCC1B, as explained in Methods. The hNKCC1A displayed an average IC_50_ for bumetanide of 0.945 µM (calculated individually from n = 7). The respective average IC_50_ for hNKCC1B was 0.842 µM (calculated individually from n = 7), which was not significantly different from the IC_50_ of bumetanide for hNKCC1A. Thus, bumetanide inhibited both hNKCC1 splice variants with about the same potency (Table 1, Fig. 3).

**Table 1:** Comparison of the potency of loop diuretics from different structural groups (see Fig. 1) to inhibit hNKCC1A and hNKCC1B.

Loop diuretic	IC_50_ for hNKCC1A		IC_50_ for hNKCC1B		Ratio hNKCC1A/ hNKCC1B
	IC_50_ (µM) (range)	Relative to bumetanide (=1)	IC_50_ (µM) (range)	Relative to bumetanide (=1)	
**1. Sulfamoyl-benzoic acid group**					
Bumetanide	0.945 (0.387–2.92)	1	0.842 (0.393–2.41)	1	1.12
Furosemide	5.15 (4.34–13.7)	0.19	5.82 (4.73–7.27)	0.14	0.885
Piretanide	3.35 (0.38–6.31)	0.28	1.66 (0.579–4.70)	0.507	2.02
**2. Non-acids with sulfamoyl group**					
Azosemide	0.246 (0.179–0.425)	3.84	0.197 (0.192–0.873)	4.27	1.25
Torasemide	6.18 (1.42–8.95)	0.153	8.19 (1.31–12.4)	0.103	0.755
Tripamide	N.E. (up to 1000 µM)	—	N.E. (up to 1000 µM)	—	—
**3. Non-sulfonamides**					
Ethacrynic acid	1678 (46.1–23990)	0.000563	3071 (309–4600)	0.000274	0.546
Ticrynafen	489 (426–1183)	0.00193	1781 (251–2703)	0.000472	0.275
**4. Non-loop diuretics with sulfamoyl group**					
Xipamide	N.E. (up to 1750 µM)	—	N.E. (up to 1750 µM)	—	—
Glybenclamide	N.E. (up to 100 µM)	—	N.E. (up to 100 µM)	—	—

For each drug, IC_50_ was at least determined in three independent experiments (range 3–7); the average IC_50_ is given as median and range of the individual IC_50_ values. N.E., not effective.

### Inhibitory potency of other loop diuretics in the hNKCC1A/B assay

In addition to bumetanide, 7 other loop diuretics (furosemide, piretanide, azosemide, torasemide, tripamide, ethacrynic acid, ticrynafen) were tested for hNKCC1A vs. hNKCC1B inhibition in the same manner as bumetanide. Measured IC_50_s are shown in Table 1. None of these drugs inhibited hNKCC1B significantly more potently than hNKCC1A. Although the average IC_50_ of piretanide for hNKCC1B was 50% lower than its IC_50_ for hNKCC1A, this difference was not statistically significant. Similarly, although ticrynafen and ethacrynic acid tended to inhibit hNKCC1A more potently than hNKCC1B (Table 1), the differences were not significant.

Interestingly, azosemide was ~4-times more potent to inhibit the two hNKCC1 variants than bumetanide. The rank order of inhibitor potencies was azosemide > bumetanide ≥ torasemide ≥ furosemide ≥ piretanide >> ethacrynic acid >> ticrynafen. Thus, loop diuretics lacking a sulfamoyl moiety (ethacrynic acid, ticrynafen) were the least potent compounds. An exception was tripamide, which contains a sulfamoyl moiety (Fig. 1) but did not inhibit hNKCC1 splice variants in concentrations up to 1000 µM. Furthermore, xipamide and glibenclamide, which contain a sulfamoyl moiety but do not act at the TALH, did not inhibit the NKCC1 splice variants. Ethacrynic acid’s inhibitory effect on hNKCC1A and hNKCC1B was highly variable between experiments, in contrast to the other tested drugs.

### Comparison of the potency of loop diuretics to inhibit hNKCC1A vs. hNKCC1B activity in the *Xenopus* oocyte heterologous expression system

**Figure 4:**
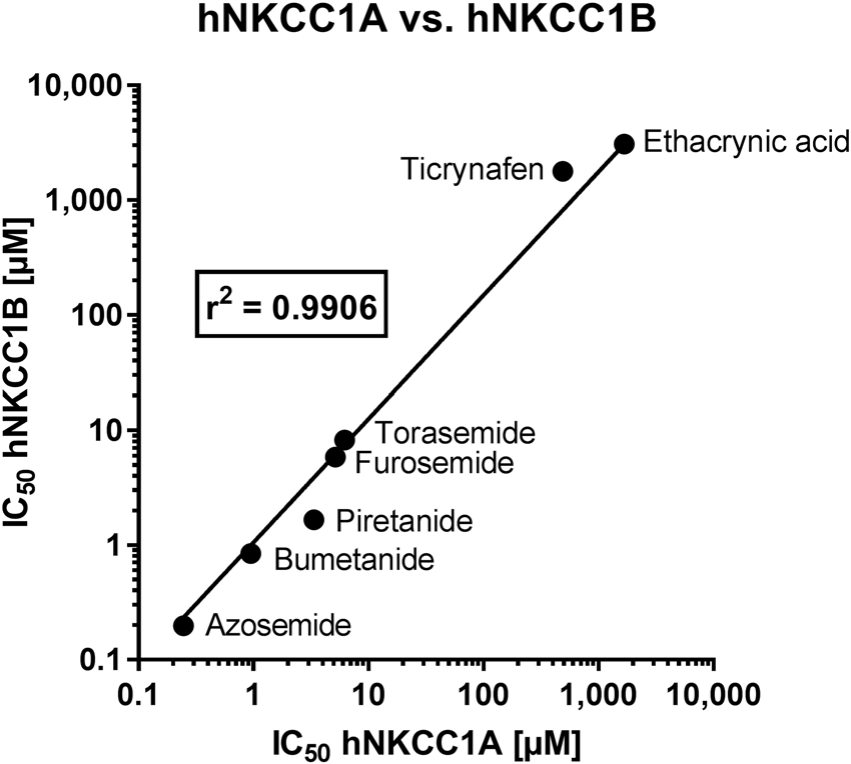
Correlation between IC_50_ for inhibition of hNKCC1A vs. hNKCC1B in the *Xenopus* oocyte assay for the loop diuretics evaluated in this study (see Table 1). Note that log data were used for correlation analysis.

To determine correlation of the IC_50_s, these values were plotted for the tested loop diuretics for hNKCC1A and hNKCC1B (Fig. 4). Correlation analysis of the IC_50_s obtained for hNKCC1A vs. hNKCC1B yielded a correlation coefficient of 0.9906, P < 0.001, indicating a strong correlation between the inhibitory potency of these compounds on the human NKCC1 splice variants tested here (Fig. 4). Only piretanide exhibited a trend for more potent inhibition of hNKCC1B.

## Discussion

To our knowledge this is the first study addressing the inhibitory action of structurally diverse loop diuretics on hNKCC1 and its major splice variants hNKCC1A and hNKCC1B. As shown in Table 2, only loop diuretics of the 5-sulfamoylbenzoic acid derivative group have been tested previously for their inhibitory activity on NKCC1. In these previous studies, benzmetanide was the most potent NKCC1 inhibitor, followed by bumetanide, piretanide and furosemide. A similar rank order of inhibitory potencies was determined for NKCC2 (Table 2). IC_50_s of bumetanide and furosemide for inhibition of NKCC1 in native cells were similar to respective IC_50_s determined in heterologous systems (Table 2). However, it has not been studied previously whether the NKCC1 splice variants NKCC1A and NKCC1B differ in their pharmacological sensitivity, whereas such studies exist for splice variants of NKCC2 (Table 2).

**Table 2:** Inhibitory potencies of loop diuretics for NKCC1 and NKCC2 based on a review of the literature.

Compound	IC_50_ (µM) NKCC2	Preparation	IC_50_ (µM) NKCC1**	Preparation	Reference
**1. Endogenous NKCCs in native cells or tissue preparations**					
Furosemide	3–7.1*	Rabbit kidney TAL; rat kidney TAL	7–23***	Turkey erythrocytes; mIMCD-K2 cells; chick cardiac cells; winter flounder intestine; rat erythrocytes and thymocytes	Palfrey *et al*.^48^; Schlatter *et al*.^23^; Frelin *et al*.^49^; O’Grady *et al*.^50^; Glanville *et al*.^51^; Hannaert *et al*.^52^
Bumetanide	0.2–0.33*	Rabbit kidney TAL; rat kidney TAL	0.25–0.6***	Turkey erythrocytes; mIMCD-K2 cells; chick cardiac cells; winter flounder intestine; rat erythrocytes and thymocytes	Palfrey *et al*.^48^; Schlatter *et al*.^23^; Frelin *et al*.^49^; O’Grady *et al*.^50^; Glanville *et al*.^51^; Hannaert *et al*.^52^
Piretanide	1–1.1*	Rabbit kidney TAL; rat kidney TAL	0.5–3***	Turkey erythrocytes; chick cardiac cells; mIMCD-K2 cells; winter flounder intestine; rat erythrocytes and thymocytes	Palfrey *et al*.^48^; Schlatter *et al*.^23^; Frelin *et al*.^49^; O’Grady *et al*.^50^; Glanville *et al*.^51^; Hannaert *et al*.^52^
Benzmetanide	?		0.05–0.3***	Turkey erythrocytes; chick cardiac cells; winter flounder intestine	Palfrey *et al*.^48^; Frelin *et al*.^49^; O’Grady *et al*.^50^
Azosemide	3	Rat kidney TAL	?		Greven^53^
Torasemide	0.3	Mouse and rabbit kidney TAL	?		Wittner *et al*.^54^
Tripamide	?	Standard clearance techniques in humans	?		Brater and Anderson^36^
Tizolemide	>100	Rabbit kidney TAL	?		Schlatter *et al*.^23^
Ethacrynic acid	5.1	Rabbit kidney TAL	?		Schlatter *et al*.^23^
Etozolin	>100	Rabbit kidney TAL	?		Schlatter *et al*.^23^
Ticrynafen(tienilic acid)	410	Rabbit kidney TAL	?		Schlatter *et al*.^23^
Indacrinone	12	Rabbit kidney TAL	?		Schlatter *et al*.^23^
Ozolinon	11	Rabbit kidney TAL	?		Schlatter *et al*.^23^
Muzolimine	>100	Rabbit kidney TAL	?		Schlatter *et al*.^23^
**2. NKCCs expressed in heterologous systems**					
Furosemide	15.1 (hA), 7.2 (hB), 10.6 (hF)	hNKCC2 isoforms (A, B, F) expressed in Xenopus oocytes	~10 (K_i_)***	hNKCC1 expressed in HEK-293 cells	Gillen *et al*.^55^; Carota *et al*.^56^
Bumetanide	0.54–4 (hA), 0.22 (hB), 0.16 (hF), 2 (mA), 0.6 (mB), 3.4 (mF)	hNKCC2 or mNKCC2 isoforms (A, B, F) expressed in Xenopus oocytes	0.16–0.28***0.68 (hA)	hNKCC1 expressed in HEK-293 cells; hNKCC1A expressed in Xenopus oocytes	Payne *et al*.^57^; Isenring *et al*.^58^; Plata *et al*.^59^; Carota *et al*.^56^; Lykke *et al*.^60^; Lykke *et al*.^29^

IC50s determined for endogenous NKCCs in native cells (or tissue preparations) or expressed in heterologous systems (Xenopus oocytes or HEK-293 cells) are separately shown. Note that several of the older studies used tissue preparations (such as TALH) without directly determining inhibition of NKCC2. For NKCC1, no data were available in the literature on isoforms of NKCC1 other than NKCC1A. Note that bumetanide is relatively selective for NKCCs, while furomedide and piretanide block KCCs at similar potencies than NKCCs^38^. Abbreviations: h, human; m, mouse; mIMCD-K2, mouse inner medullary collecting duct cell line; TALH, thick ascending limb of Henle. *NKCC2 isoforms not differentiated; **Activated state; ***NKCC1 isoforms not differentiated.

In the present study, 8 loop diuretics of the 3 structurally different groups shown in Fig. 1 were used to evaluate structure-activity relationship of inhibition of hNKCC1A vs. hNKCC1B. Based on their structure, these drugs may be also differentiated into acidic and basic compounds. Furthermore, two sulfamoyl derivatives that do not act on NKCC2 (Table 2), i.e., the thiazide diuretic xipamide and the antidiabetic drug glibenclamide, were included in our experiments. As expected, only loop diuretics inhibited the hNKCC1 splice variants, whereas xipamide and glibenclamide were inactive. However, marked differences were observed across the diverse loop diuretics.

Azosemide was the most potent inhibitor of hNKCC1, inhibiting both splice variants with about the same efficacy. Azosemide lacks the carboxylic group of the 5-sulfamoylbenzoic acid derivatives (Fig. 1), demonstrating that this carboxylic group is not needed for potent inhibition of NKCC1. Clinically, azosemide has about the same diuretic potency as furosemide, but both drugs are clearly less potent than bumetanide^30^, so the high potency of azosemide to inhibit the hNKCC1 splice variants was unexpected. In contrast to the short-acting diuretic bumetanide, the long-acting azosemide is not a carboxylic acid, so that its tissue distribution should not be restricted by a high ionization rate. However, it is highly bound to plasma proteins^31^, which might limit its penetration into the brain. Indeed, in a study in which the tissue distribution of azosemide was determined 30 min following i.v. administration of 20 mg/kg in rats, brain levels were below detection limits (0.05 µg/g^32^).

Other non-acidic loop diuretics that were evaluated in the present study were torasemide and tripamide. Torasemide potently inhibited both hNKCC1A and hNKCC1B, while tripamide was ineffective up to 1000 µM. Clinically, torasemide (or torsemide) is a long-acting diuretic that is about 3 times more potent than furosemide^30^. Torasemide is highly bound to plasma proteins, which may explain its low apparent volume of distribution^33^. Tissue distribution studies following i.v. administration of 10 mg/kg torasemide in rats found that the brain:plasma concentration ratio 30 min after injection is only 0.00024^34^, which is even lower than the brain:plasma concentration ratio of bumetanide in rats (~0.01–0.02^35^). The poor brain penetration of azosemide^32^ and torasemide^34^ indicates that, similar to bumetanide^12^, in addition to high plasma protein binding, active efflux at the BBB restricts brain entry of these drugs.

Tripamide, which belongs to the same group as torasemide (Fig. 1), has been shown to act as a loop diuretic^36^, but no IC_50_ for NKCC2 was available from the literature (Table 2). Tripamide is less well characterized than most other loop diuretics and standard clearance techniques indicate that it may also affect more proximal nephron sites^36^.

Bumetanide was the second most potent NKCC1 inhibitor of the series of loop diuretics studied here, inhibiting both splice variants with the same potency. The two other members of the 5-sulfamoylbenzoic acid derivatives group, furosemide and piretanide, were also highly potent inhibitors of the two hNKCC1 splice variants with IC_50_s in the low µmolar range. While furosemide was without any clear preference for either variant, piretanide tended to inhibit hNKCC1B twice more potently as hNKCC1A, although the difference was not statistically significant.

The two members of the non-sulfonamide group, ethacrynic acid and ticrynafen, were markedly less potent in inhibiting the hNKCC1 splice variants than loop diuretics containing a sulfamoyl moiety, with the exception of tripamide. This may indicate that the sulfamoyl moiety is a prerequisite for potent inhibition of NKCC1. Interestingly, both compounds were moderately more potent in inhibiting hNKCC1A than hNKCC1B. While the low potency of ticrynafen to inhibit hNKCC1 splice variants was in line with its low potency to inhibit NKCC2, this was not the case with ethacrynic acid, which is a potent diuretic drug (Table 2) with a clinical efficacy similar to that of furosemide^30^.

Overall, the present data indicate that loop diuretics have no significant hNKCC1A/hNKCC1B selectivity, when hNKCC1-mediated transport is measured at the transporters’ activated state. Full-length NKCC1 (NKCC1A) comprises ~1200 amino acids and has a molecular weight of 130–132 kDa^37^. The predicted three-dimensional topology of NKCC1 shows 12 α-helical transmembrane (TM) domains which are flanked by hydrophilic amino- and carboxyl-terminal regions^38^. The shorter NKCC1B splice variant, lacking exon 21, was first detected by partial cloning in mouse brain tissue^19^. Exon 21 encodes 16 amino acid residues from the carboxyl (C) terminus of the NKCC1 protein. The 16 amino acids fragment was shown to contain a dileucine motif as a basolateral sorting motif, therefore targeting NKCC1A to the basolateral membrane of epithelia^39^. The absence of exon 21 in NKCC1B does not appear to affect NKCC1 function^37^ and, as demonstrated by the present data, susceptibility to inhibition by loop diuretics. Interestingly, exon 21 of NKCC1A is also absent from NKCC2, which is involved in the apical expression of the latter transporter, although there are other important signals in the C-terminus of NKCC2 that take it to the apical membrane^39^. It is generally thought that loop diuretics bind in the translocation pocket of NKCC and that the binding site may be near the intracellular end of the pocket^40–44^.

One may thus argue that the outcome of this study was essentially predictable given that NKCC1B is identical to NKCC1A except for the exclusion of a single exon in the C-terminus. In particular, NKCC2 also lacks this exon and is as sensitive to bumetanide as is NKCC1A (Table 2). Thus, loop diuretics may not be an ideal starting point to synthesize NKCC1B-selective inhibitors. However, as demonstrated by azosemide, potency to inhibit NKCC2 (Table 2) did not predict its high potency to inhibit hNKCC1A/B in the present study.

Based on the tissue distribution and cellular localization of NKCC1 and its splice variants^18,37,38,45,46^, a NKCC1B-selective inhibitor may lack ototoxic effects while enabling inhibition of neuronal NKCC1. However, it may be difficult to develop NKCC1B-selective inhibitors because the only difference between NKCC1A and NKCC1B is the lack of the small exon 21, a region that is very poorly conserved, predicted to be unstructured, and seems unlikely to be involved in an important way with either ion translocation or diuretic inhibition and probably not to a significant extent to the phosphorylation-activation mechanism^38^. One feasible plan to tackle the goal would be a high-throughput screening (HTS) assay for the splice variants of the hNKCC1 cotransporter similar to the HTS approach recently described for hNKCC1 expressed in human embryonic kidney (HEK) cell line^47^. The latter cell-based Rb^+^ flux assay has been used to screen for NKCC1 inhibitors in a focused library of 1450 compounds, followed by a full HTS of 1.2 million compounds, using the Ion Channel Reader (ICR) technology for detecting intracellular concentration of Rb^+^ in cell lysates^47^. IC_50_ for bumetanide (1.16 µM) determined by the latter technology was similar to the bumetanide IC_50_s reported here, but none of the blind compounds reported by Gill *et al*.^47^ reached the potency of azosemide determined in the present study.

In conclusion, the main findings of the present study on structure-activity analyses of 10 chemically diverse diuretics are that (1) none of the examined compounds were significantly more effective to inhibit NKCC1B than NKCC1A, and (2) azosemide was more potent than any other diuretic, including bumetanide, to inhibit the two NKCC1 variants. The latter finding is particularly interesting because, in contrast to bumetanide, which is a relatively strong acid (p*K*a = 3.6), azosemide is not acidic (p*K*a = 7.38), which should favor its tissue distribution by passive diffusion. Lipophilicity (log*P*) of the two drugs is in the same range (2.38 for azosemide vs. 2.7 for bumetanide). Furthermore, azosemide has a longer duration of action than bumetanide, which results in superior clinical efficacy^26^ and may be an important advantage for treatment of brain diseases with abnormal cellular chloride homeostasis.

## Electronic supplementary material

Supplementary Fig. 1
