# Supplementary material for: Azosemide is more potent than bumetanide and various other loop diuretics to inhibit the sodium-potassium-chloride-cotransporter human variants hNKCC1A and hNKCC1B
Source: Sci Rep. 2018 Jun 29;8:9877. doi: 10.1038/s41598-018-27995-w (PMC6026185; doi:10.1038/s41598-018-27995-w)
Supplement: Supplementary file 1 — Supplementary Fig. 1 [file 41598_2018_27995_MOESM1_ESM.pdf]

Azosemide is more potent than bumetanide and various other loop diuretics to inhibit the sodium-potassium-chloride-cotransporter human variants hNKCC1A and hNKCC1B

Philip Hampel[1,2§], Kerstin Römermann[1§], Nanna MacAulay[3], Wolfgang Löscher[1,2*]

[1]Department of Pharmacology, Toxicology and Pharmacy, University of Veterinary Medicine Hannover, Germany

[2]Center for Systems Neurosciences Hannover, Germany

[3]Department of Neuroscience, University of Copenhagen, Denmark

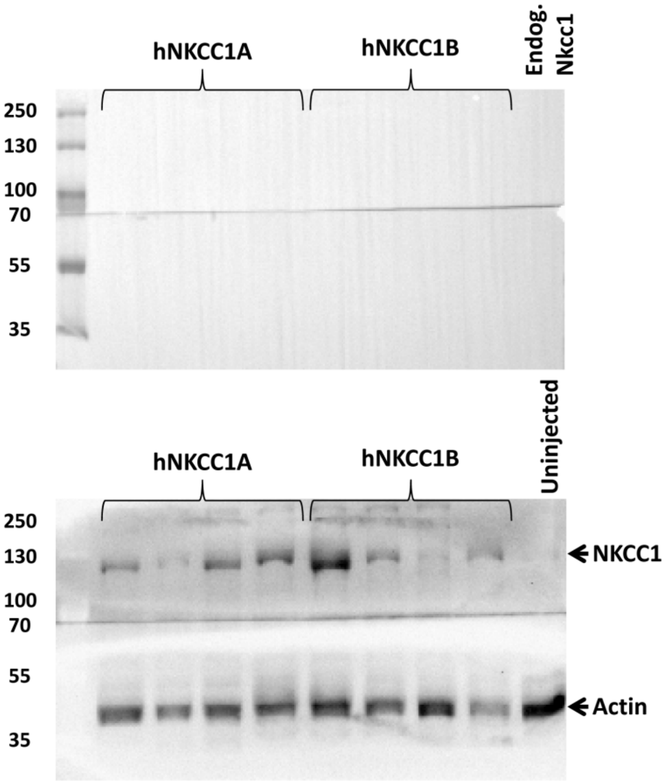

Supplementary Fig. 1.

Representative Western blots of hNKCC1A and hNKCC1B protein expression in injected oocytes as well as endogenous NKCC1 in uninjected oocytes. All membranes displaying β-actin and NKCC1 were cut at 70 kDa prior to the antibody incubation and subsequently reassembled for image acquisition. Marker and samples shown in one blot were all separated through the same SDS-PAGE. Figure 2A displays an overlay of marker and protein bands. This overlay was assembled from two individual images, as the marker is only visible using transmitted light whereas other settings are required for detection of the protein bands. Hence, the original images of the marker (A) and the protein bands (B) are shown separately here.
